# Transparent Colloids of Detonation Nanodiamond: Physical, Chemical and Biological Properties

**DOI:** 10.3390/ma16186227

**Published:** 2023-09-15

**Authors:** Stepan S. Batsanov, Sergey M. Gavrilkin, Dmitry A. Dan’kin, Andrei S. Batsanov, Alexander V. Kurakov, Tatiana B. Shatalova, Inna M. Kulikova

**Affiliations:** 1 National Research Institute for Physical-Technical Measurements, Mendeleevo 141570, Russia; gavrilkin@vniiftri.ru; 2 Fritsch Laboratory Instruments, Moscow Branch, Moscow 115093, Russia; dankin_dima@bk.ru; 3 Chemistry Department, Durham University, Durham DH1 3LE, UK; a.s.batsanov@durham.ac.uk; 4 Biological Faculty, Moscow State University, Moscow 119991, Russia; kurakov57@mail.ru; 5 Chemistry Department, Moscow State University, Moscow 119991, Russia; shatalovatb@gmail.com; 6 Institute of Mineralogy, Geochemistry and Crystalchemistry of Rare Elements, Moscow 121357, Russia; imkulikova@gmail.com

**Keywords:** detonation nanodiamond, suspension, dielectric-permittivity, nitrogen fixation, ammonium selitre

## Abstract

Aqueous suspensions (colloids) containing detonation nano-diamond (DND) feature in most applications of DND and are an indispensable stage of its production; therefore, the interaction of DND with water is actively studied. However, insufficient attention has been paid to the unique physico-chemical and biological properties of transparent colloids with *low* DND content (≤0.1%), which are the subject of this review. Thus, such colloids possess giant dielectric permittivity which shows peculiar temperature dependence, as well as quasi-periodic fluctuations during slow evaporation or dilution. In these colloids, DND interacts with water and air to form cottonwool-like fibers comprising living micro-organisms (fungi and bacteria) and DND particles, with elevated nitrogen content due to fixation of atmospheric N_2_. Prolonged contact between these solutions and air lead to the formation of ammonium nitrate, sometimes forming macroscopic crystals. The latter was also formed during prolonged oxidation of fungi in aqueous DND colloids. The possible mechanism of N_2_ fixation is discussed, which can be attributable to the high reactivity of DND.

## 1. Introduction

The synthesis of nanoscale diamond powder by detonating explosives in the absence of air is a unique example of when, as a result of fundamental research, the expensive and environmentally harmful disposal of explosives after the expiration of their safe storage time (by incineration or dissolving in water) has been turned into a profitable industrial production of nanomaterials with huge prospects for their use in science, various technologies, and for biomedical purposes. 

**Figure 1:**
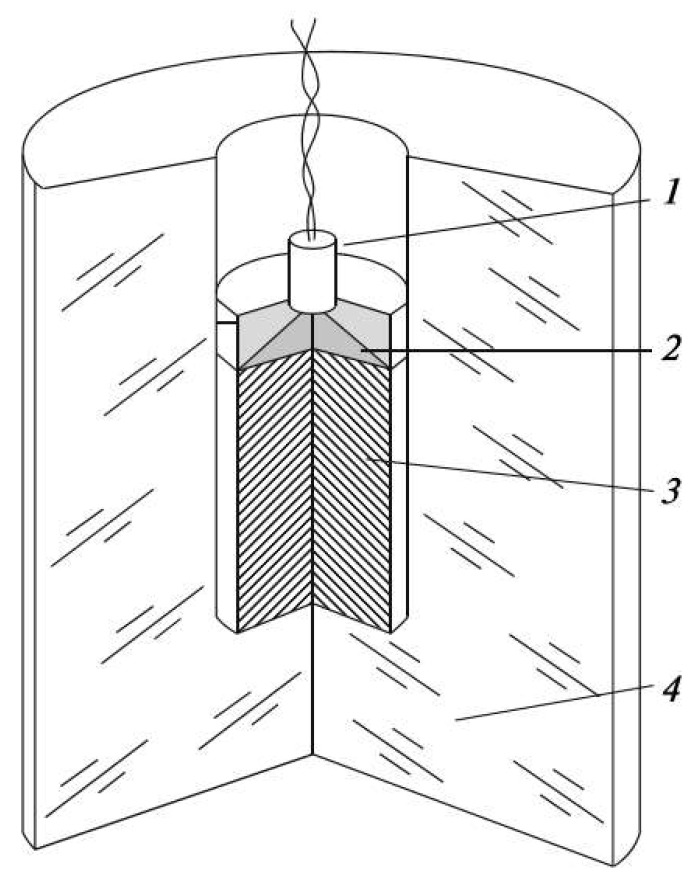
Detonation experiment set-up: (1) detonator, (2) plane wave generator, (3) alloy of trinitrotoluene (TNT) and cyclotrimethylene trinitramine (RDX), (4) ice shell.

DNDs, with typical crystal sizes of 4–5 nm, are produced by detonating high explosives enclosed in an ice shell (Figure 1). The latter allows for rapid cooling (quenching) of the detonation products, avoiding the annealing of diamond into graphite by the residual heat of the explosion. The detonation soot is then subjected to the intense oxidation to remove the graphite component and isolate the DNDs. 

The first detonation synthesis of nanodiamond published in [1,2] initiated a lot of work on the study and application of DND in various fields of science, technology and biomedicine; in many of these fields (stable electrodes, water absorbent materials, galvanic processes, preparation of some polymer-DND composites, biosensors, drug delivery), it functions in the form of colloidal suspensions in water [3,4,5]. The production of DND also typically involves its aqueous suspensions as an indispensable stage. Therefore, understanding their organization and properties have paramount importance, and it has become the subject of numerous studies. However, most studies, publications and patents were focused on concentrated aqueous suspensions or slurries of DND, and insufficient attention was paid to the structure, physico-chemical and biological properties of dilute colloids. Therefore, the present review focuses on the latter. 

Aqueous DND suspensions with various carbon contents (wt%): 0.007–0.2 [6], 0.05 [7], 0.2 [8,9], 0.6–3.1 [10], 1–8.6 [11], 1–10 [12] and 10–50 [13] have been reported. They are, as a rule, dark or brown in color, but those obtained after centrifugation are colorless and transparent [7]. In the authors’ laboratory, after removing graphite from the detonation soot by oxidizing with HClO_4_, the suspension formed by using sonication and subsequent centrifugation at 15,000 rpm also was converted into a clear colorless suspension containing ca. 0.01–0.1 wt% of DND, henceforth referred to as ‘diamond water’ (DW). This liquid displays a variety of unique and puzzling properties, some of which hold technological promise; please refer to [14] for additional information. However, data on DW are still incomplete, and certain aspects remain unexplained and even contradictory, necessitating further research. 

**Figure 2:**
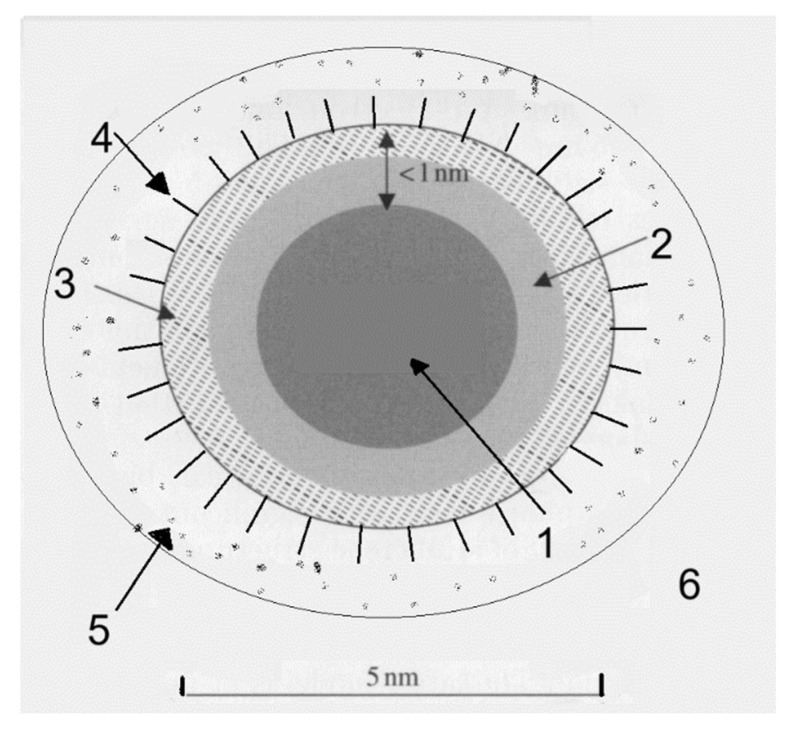
Scheme of a DND particle in aqueous colloid: (1) diamond core, (2) layer of distorted diamond, (3) outer layer of amorphous sp^3^ carbon or sp^2^ carbon, (4) surface-terminating groups (COOH, OH, etc.), (5) confined water, (6) bulk water.

Colloidal suspensions are complex systems. This complexity depends on the materials, their concentrations, the particle sizes, and various atomic interactions, such as electrostatic repulsion, depletion forces, van der Waals attraction and Brownian motion, with the coagulation of particles being another important effect. Furthermore, a DND particle itself has a complex, variable and still-debated structure (Figure 2) [15,16,17,18] and should be regarded as a giant molecule rather than a simple fragment of a bulk diamond structure. According to X-ray diffraction (XRD) and transmission electron microscopy (TEM) data, it typically comprises a diamond core ca. 3 nm in diameter, surrounded by ca. 1 nm thick shell of amorphous sp^3^ (diamond-like) carbon. The surface, however, cannot consist of the latter without allowing ‘dangling’ bonds and is either reconstructed into well-ordered chains of π-bonded carbon atoms (so-called ‘2 × 1’, or Pandey reconstruction) [19], converted into a complete graphitic shell [20] or is terminated by other groups or atoms. The as-produced and oxidatively purified DND is terminated by oxygen-containing functional groups (hydroxyl, carbonyl, carboxyl, lactone, nitro, etc.), which under reductive conditions can be replaced by hydrogen atoms (C–H termination). Thus, DND is not pure diamond, and normally not even pure *carbon*, typically containing 10–15% of other elements. 

## 2. Structure and Physical Properties of DND Colloids 

### 2.1. Structure of DND Colloidal Solutions

Suspensions with a high carbon content are typical heterogeneous systems, containing a mixture of liquid and solid phases, which, after intensive centrifugation, turn into homogeneous transparent (to the human eye) solutions containing particles less than 1 μm in size. Indeed, studies of these liquids by the dynamic light scattering (DLS) method show that the particle-size distribution in them varies from tens to hundreds of nanometers [7,8,12,21]. Since the particles in colloidal solutions are much larger than the size of the DND grain (a few nm in size), it is natural to assume that the primary particles have a very strong tendency to agglomerate through covalent bonding as well as coherent interfacial Coulombic interactions [8,13,22]. 

However, this agglomeration is not just a close packing of primary particles, as was presumed initially. Firstly, agglomerated DND has specific surface area of 180 to 300 m^2^g^−1^ from Brunauer–Emmett–Teller (BET) adsorption–desorption data, which corresponds to effective particle sizes of 6–10 nm, almost as small as the primary particles [3,4,5]. Secondly, agglomerated DND contains mesopores with the diameters larger (15–18 nm) [23] or at least as large (4.5 nm) [24] as the primary particles themselves. DND powder is highly hygroscopic even though its surface is hydrophobic [24]. The confined water is firmly connected to the diamond surface (the last portions of water are removed only at 800 °C [25]), and DND which has absorbed its own weight of water still flows as well as the dry powder [23]. These facts, as well as TEM and scanning electron microscopy (SEM) observations, can be explained [14,20] by primary DND particles connecting through certain vertices or faces to form a porous framework, with water largely confined inside the pores. However, one would expect such frameworks to be rather fragile, which makes their persistent resistance to de-aggregation puzzling. 

**Figure 3:**
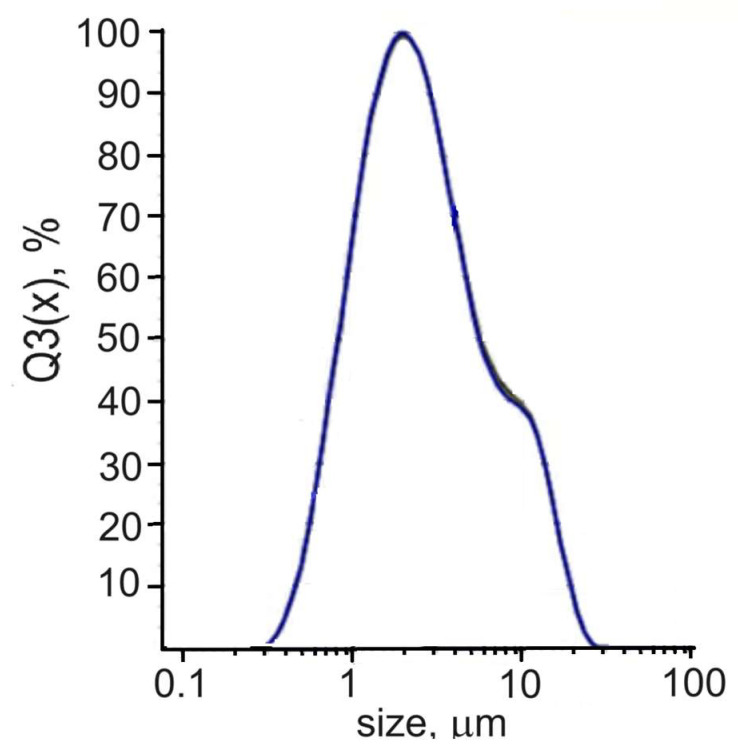
Sizes of grains in dry DND powder measured by SLS method.

In the examination of dry DND powder using static light scattering (SLS) as depicted in Figure 3, a consistent range of particle sizes was observed, spanning from 0.3 to 24 μm. The average particle size measured 3.9 μm, with a peak at 2 μm and a recognizable shoulder evident at 10 μm. Hence, it can be assumed that the disintegration of large particles occurs in an aqueous solution. However, given the strong bonds between the diamond grains, most likely only the smallest particles pass into the aqueous solution; this alternative is confirmed by the results of electro-physical measurements (see below).

It was shown by Fabry–Perot interferometry [26] that colloidal particles in DW with average diameters varying from 106 to 854 nm have the refractive indices of 1.74 to 1.79 (cf. *n* = 2.1 in dry DND), which corresponds to the 14% water content within these particles, and the density of particles (*ρ*_p_) in DW equaled 2.2 g/cm^3^. This fact is consistent with the strength of bonding between water molecules and the surface of DND [25] and the high hygroscopicity of the latter [23,24].

Due to diffusion, particles of DND in stable aqueous colloidal solutions should be evenly distributed in the liquid at distances depending on their concentration and sizes. Table 1 provides data on the diameters (*D*) of DND particles in water, their corresponding volumes (*V*_p_) and the concentrations (*C*) of two samples, calculated as 0.01/2.2 = 0.045 and 0.1/2.2 = 0.45 vol% (wt%/*ρ*_p_). Additionally, the table presents the water volume per particle (*V*_w_ = $\frac{100}{C}$*V*_p_) and the radii of water spheres (*R*_w_) surrounding DND particles within DWs (not to be confused with the size of the particle itself, as shown in Figure 3). An investigation of mature DW by luminescent microscopy [27] showed that DND particles formed a quasi-lattice pattern with fairly uniform nearest separations of 2 to 3 μm. These distances agree with the *R*_w_ values estimated above. Note that the particles’ own size is smaller by an order of magnitude and was near the limit of the microscope resolution.

Thus, the sizes of DND particles vary in the succession: in dry powder > in an aqueous colloid > in shock-wave synthesis [28]. Consequently, whether ‘agglomeration’ or ‘disintegration’ of grains will occur primarily depends on their predecessors, but in reality, an independent solution to this problem is also possible. Indeed, the clear colorless colloid (DW) prepared from DND contained ca. 0.01–0.1 wt% of DND. Note that during preparation of DW, only ca. 1% of the DND dispersed in water remained in suspension after spontaneous precipitation and centrifugation. To ensure that this fraction was representative of the whole, some of the DW was evaporated and the dry residue subjected to elemental and X-ray powder diffraction analyses, which confirmed its identity. Assuming a Gaussian distribution of particle sizes, there should be a tiny fraction of very small particles present, which would be the first to be extracted by water. In [23], it was found that re-moisturizing a DND sample previously that was treated by water and dried gives a smaller electro-physical effect compared to a fresh sample. The decrease can be attributed to the loss of the smallest DND particles, which are leached out during first water treatment or lifted into the air during drying. Such particles were in fact found in the exit cold trap and identified as DND by electron diffraction and microscopy ([23], see ESI). Measurements of the DND particle suspended in CCl_4_ and dimethylformamide showed sizes of 10 and 36 nm, respectively [21], and ca. 100 nm in toluene (cf. 400 nm in water, using the same DND sample) ([27] see ESI). Since the concentrations of DND particles in transparent colloids vary from 0.01 to 0.1 wt% [8,23,29], leaching of the smallest particles by liquids, which make up the same proportion in nanodiamond powder, is an important contribution to deagglomeration along with mechanical crushing [8,12], chemical modification of the DND surface [30] and the removal of surface layers of particles by oxidation [31].

The stability of colloidal solutions depends on the size of the particles that will precipitate over time in accordance with Stokes’ law. The precipitation time (*τ*_prec_) of a particle is

(1) $$\tau_{\mathrm{prec}}=\frac{18h\eta }{D^{2}\;\left(\rho -\rho_{0}\right)g}$$

where *D* is the particle size, *ρ* is its density, *h* is the height of its descent, *η* and *ρ*_o_ are the viscosity and density of the medium (in this case, water) and *g* is the gravitational acceleration. Equation (1) predicts that DND agglomerates with *D* = 1, 0.5 and 0.1 μm would precipitate in 4 h, 16 h and 17 days, respectively, while 10 μm ones in just 2.5 min. On the contrary, primary DND particles of 5 or 10 nm, if dispersed, will remain suspended practically indefinitely (*τ*_prec_ = 19 and 4.8 years, respectively), although their density is higher than that of agglomerates, which are a mixture of porous DND frameworks and water. This, of course, is a crude estimate, precipitation being a complicated process. To the authors’ knowledge, no direct experimental measurement of this interesting effect has been reported so far; however, it is noteworthy that hydrosols of deagglomerated primary DND particles proved remarkably stable and did not show any turbidity or precipitation of solid over several years ([14] and references therein).

### 2.2. Physical Properties of DND Colloidal Solutions: Static

DW is slightly acidic. Remarkably, its pH of 6.25–6.77 does not change after long exposure to atmospheric air, i.e., to atmospheric CO_2_, whereas pure water in equilibrium with the latter at room temperature acquires pH = 5.6. Even the DW of second and third extraction (vide supra), with relatively low dielectric permittivity (DP, or ε), shows similar behavior.

The transparent colloidal solutions of DND are the most convenient objects for electro-physical measurements, which are very sensitive to changes in the atomic and electronic structure of homogeneous media. For example, in [32], it was established that the dielectric permittivity of DND suspensions in *polar* organic solvents are higher than those of the solvents themselves, increasing sympathetically with the dipole moment of the solvent, as shown in Table 2. 

In DWs, the effect is much greater and shows the trend of change in the dielectric constant (ε) and the resistivity (R) with the concentration of DND, as shown in Table 3.

Notwithstanding the low concentration of DND, the colloids showed giant ε, varying for different preparations from 10^4^ to 10^6^ at AC of 1 kHz (cf. 78 for pure water). Although dry DND has higher ε (measured ε = 21 [23]) than bulk diamond (ε = 5.75), this is nowhere near enough to account for this effect. It is well known that liquids containing ionic species show very high (apparent) ε at low-frequency AC, because mobile ions have time to move towards the electrodes and create double electric layers at their surfaces [33,34]; correcting for these electrode polarization effects is a complicated problem, which is not fully resolved [35]. This applies even to highest purity water, where the dissociation into OH^−^ and H^+^ always occurs. Contamination of water with ion-generating atmospheric gases (especially CO_2_), which is very difficult to avoid, amplifies the effect. Obviously, the same can be expected from DND particles prepared under oxidative conditions, which has ionizable surface-terminating groups (carboxylic and hydroxylic). However, electrode polarization effects only became noticeable at frequencies lower than 1 kHz; above this point, the curve was practically flat [33,34]. Therefore, the dielectric permittivity measured at 1 kHz can be regarded to be reasonably free of polarization errors and all fixed-frequency measurements are carried out (vide infra) at this frequency.

**Figure 4:**
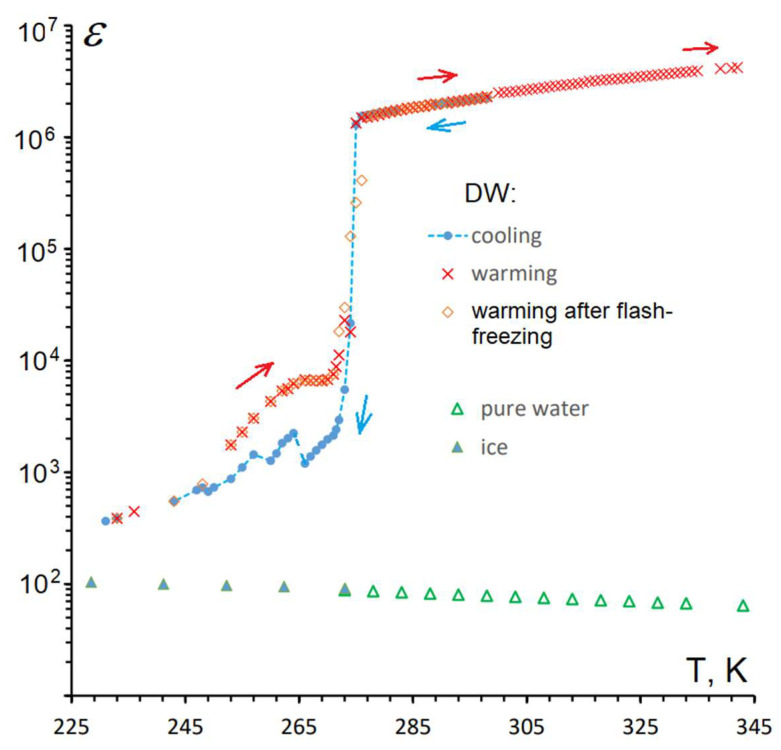
DP as a function of temperature of liquid and frozen DW, compared to pure water. Arrows show the direction of temperature change.

Numerous studies of electro-physical properties in hydrated powders, aqueous suspensions and colloids [36,37,38,39,40,41,42,43,44] have demonstrated both the conductivities and the dielectric permittivities of such heterogeneous systems are greatly enhanced by orders of magnitude compared to the corresponding dry solid or/and pure water. This effect is widely utilized in geological surveying [45]. The magnitude of effect is demonstrated by Figure 4, made using the authors’ measurements of the temperature changes of ε in liquid and frozen DW between 342 and 231 K.

### 2.3. Physical Properties of DND Colloidal Solutions: Dynamic

An obvious question is whether the colloid remains spatially homogenous during (and after) freezing. Indeed, on slow (48 h) freezing of DW, DND particles were observed to concentrate in one end of the frozen sample ([27], ESI). However, in the present experiment, the much faster cooling rate of the capacitor cell should not allow time enough for such segregation. Indeed, cooling DW at the rates of 0.5, 1 and 1.5 K/min gave essentially the same results. A DW sample frozen at 0.5 K/min and a flash-frozen one showed practically identical behavior on warming up to 277 K, and above this point the warming curves of both practically coincided with the cooling curve of the original DW (see Figure 3). In our opinion, this proves that the distribution of DND particles in frozen DW in this experiment remains sufficiently homogenous. When DW is allowed to evaporate naturally in an open vessel, its DP generally rises as the overall DND concentration increases, but the rise is not monotonicbut shows quasi-periodic fluctuations ([27], ESI). Similar fluctuations of DP were shown by DW samples kept in sealed vessels, where the concentration remains constant. These fluctuations can be explained by periodic precipitation (according to Stokes’ law, see Section 2.1) of the largest particles (agglomerates), which depletes the DND concentration in the volume of DW. The increase in concentration due to evaporation obviously facilitates the agglomeration and hence the precipitation. 

**Figure 5:**
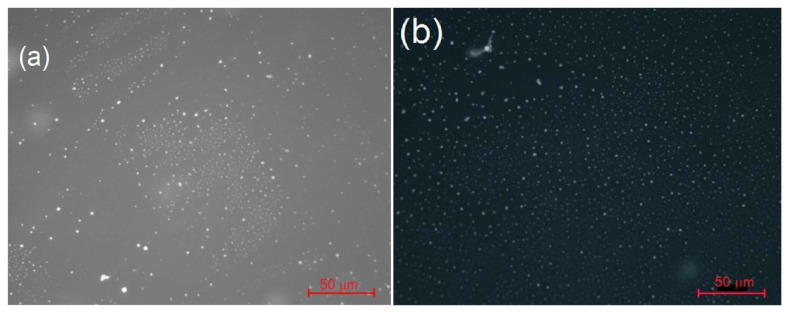
Luminescent microscopy images of DW (layer ca. 0.2 mm deep on a glass slide; resolution 0.23 µm/pixel): (**a**) freshly prepared, (**b**) after 4 h, on the same scale. Reproduced with permission from ref. [26], ©Royal Society of Chemistry.

The fluorescent properties of DND make it possible to monitor the distribution of particles in DW in real time. Figure 5 shows drops sampled from the upper layer of freshly prepared DW and the same ‘matured’ over 4 h [27]. In the latter, both the spatial distribution and the sizes of particles are obviously more uniform: the range of closest interparticle distances narrowed from 0.55–10 µm (mean 0.75 µm) to 0.7–5.5 µm (mean 0.90 µm) and larger particles have mostly disappeared. Thus, the periodic change in ε in DW corresponds to the particle lattice rearrangement, i.e., its phase transition.

According to standard DLVO theory, interfacial effects occur at distances of a few nm from surfaces of solids [46], but the experimental studies revealed interactions extending many orders of magnitude further [47,48]. The *increasing* changes of NMR relaxation times in highly diluted aqueous solutions [49] also suggests very long-range interactions. 

**Figure 6:**
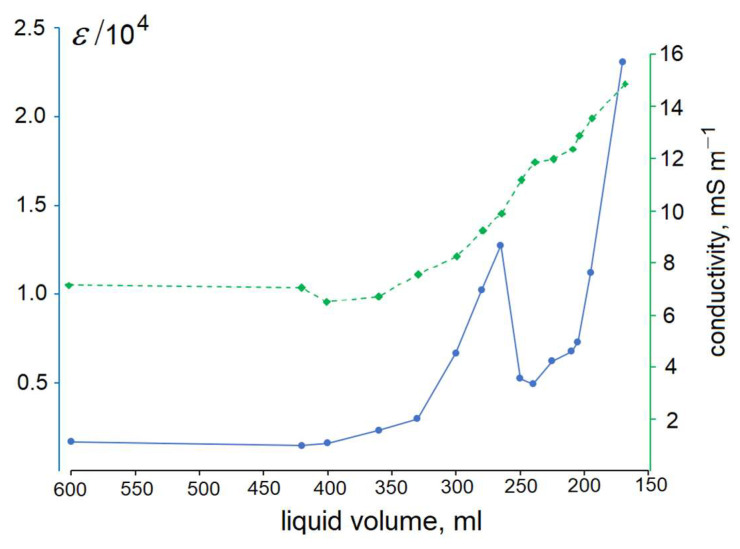
Evolution of DP (blue, left scale) and electric conductivity (green, right scale) during evaporation of DW prepared from hydrogen-free DND.

Non-monotonic changes of permittivity and conductivity of DW occur not only during its evaporation (Figure 6), but also during dilution. Remarkably, only after 10^8^ to 10^10^-fold dilution, ε(DW) approached that of pure bulk water. Also, somewhat paradoxically, the first three dilutions (i.e., up to 1000-fold) caused successive increases of the DND particle sizes spread (according to DLS measurements), as Table 4 shows; after that, larger particles began to precipitate, which causes the rearrangement of the lattice [26]. Enlargement of DND particles after a 100-fold dilution was reported also in [43]. 

The above-mentioned new experimental data, along with previously known facts [50,51,52,53], allow us to make an unambiguous conclusion that as a result of the interaction of water with powders or porous solids, the DP of water should sharply increase. However, recent studies by means of electrostatic or atomic force microscopies (EFM or AFM) [54,55,56,57] showed the DP of confined water (CW) to be much *lower* than that of bulk water (BW). If this is so and the structure (and hence the electro-physical properties) of the solid component remain unaltered, then where does the giant DP of the water–solid combination comes from? According to theoretical models [58,59,60,61,62,63,64,65,66,67,68,69,70,71,72], CW is structured in such a way that molecular dipoles are oriented parallel to the surface of the adsorbing solid. Therefore, its DP in becomes strongly anisotropic, ranging from 4 or 5 in the direction normal to the surface [60,65], to 750 [43] in the parallel direction. EFM or AFM measure only the former component, while the macroscopically observed DP of a heterogeneous mixture with randomly oriented water–solid interfaces is the much larger directionally averaged value. While this explains the above paradox, it should be noted that the predicted anisotropy of CW is difficult to reconcile with calorimetric evidence [73] that CW remains liquid at very low temperatures (150–230 K) due to break-up of hydrogen bonds. 

The observed values of ε(DW) are quite unprecedented. Structural changes in aqueous colloids are detectable due to very high sensitivity of the dielectric method: in DW with carbon content within hundredths of one per cent, ε reaches 10^5^–10^−6^ (at 1 kHz) at room temperature and experiences a significant increase upon heating. This effect is one of greatest complexity and still imperfectly understood; however, we always observed the direct dependence of permittivity on conductivity of DW, according to the known relation

(2) $$\varepsilon (\omega )=\varepsilon \prime (\omega )+j\varepsilon \prime \prime (\omega )=\varepsilon_{r}(\omega )\varepsilon_{0}+j\;\frac{\sigma \;\left(\omega \right)}{\omega }$$

where ω is the characteristic frequency and σ is the conductivity. Thus, the giant permittivity of DW is linked with its electric conductivity. Another, and fascinating, explanation is DND particles causing structural changes in the surrounding water, extending far beyond the double electric layer. It is well known that in ordinary ice (ice I) each molecule (i.e., its oxygen atom) donates two and accepts two hydrogen bonds, while in liquid water the number of hydrogen bonds per molecule is reduced, allowing a less rigid but more dense packing. The anomalous behavior of water near the freezing point, particularly its maximum density at 277 K, is often explained by transformations between de-structured H-bonded water and structured H-bonded water, or even by formation of nano-ice particles [74]. DW having lower density than pure water (although the added DND is heavier) but the sound velocity (which is usually proportional to density) in DW is higher than in pure water. In both properties, DW differs from pure water in the same sense (albeit to a much smaller extent) as ice. The temperature dependence of the permittivity of liquid and frozen DW (Figure 4) shows the most drastic change near 277 K, *not* at the freezing point. 

## 3. Chemical Properties of DND Colloids

DND particles have a unique chemical activity due to their small sizes. This so-called ‘size effect’ is common to other nano-solids and manifests itself in lower surface tension, melting point, heat capacity and atomization energy [75,76,77]; it is due to nanoparticles having a large fraction of surface atoms with lower coordination numbers. Both experiment and theory show that the atomization energy of nanoparticles decreases with the decrease in their size in a nearly linear fashion, in accordance with Equation (3) [78]

(3) $$E_{p}=\frac{N_{p}}{N_{b}}E_{b}$$

where *E*_p_ and *E*_b_ are the atomization energies, and *N*_p_ t and *N*_b_ are the average coordination numbers of atoms in the particle and in the bulk, respectively. The *E*_p_ calculated by Equation (3) for some elemental solids (Table 5) [77], are lower than *E*_b_ by tens of kJ/mol, which should substantially enhance their reactivity. Indeed, nanosized C, Si and Ge react with water under ambient conditions, substituting hydrogen [77]

(4) A + 2H_2_O = AO_2_ + 2H_2_

For Cu, the linear decrease in *E*_p_ with *N*_p_, and a lower equilibrium potential for reacting nano-state with water were predicted by DFT calculations [79], while thermodynamic analysis [80,81] shown that under certain conditions nanosized Pt can form PtO in water.

Bradac and Osswald [82] showed that the rate of oxidation of DND crystals increases with the decrease in their size (in the 2–20 nm range) and determined the critical size (1.60 ± 0.35 nm) below which DNDs are oxidized instantaneously in air and, therefore, cannot exist. Crystallographic calculations [83] show that in diamond particles with sizes 1 and 2 nm the average coordination numbers are equal to 2.4 and 3.1, respectively; i.e., these crystals, strictly speaking, no longer have the diamond structure.

In 2011, it was observed that in vials of DW exposed to atmospheric air at room temperature, after periods of time ranging from several days to several months, cloudy and then cotton wool-like entangled light-brown or grey-colored fibers of up to 1 μm in diameter (‘diamond water fibers’, DWF) formed and gradually increased in size, ultimately reaching several mm in length. In identical vials of pure water kept alongside, no fibers were formed; moreover, if transplanted there, the fibers failed to develop and soon disintegrated [21]. Authors of [84] reported details of forming DWF, its composition and properties. Table 6 shows the results of studies on several samples of DWF extracted from DW after different times (decreasing from top to bottom) of exposure to air. As one can see, they have a very complex composition with a high oxygen content and, accordingly, low values of density and refractive indices. XRD study of DWF demonstrates a halo [21], which is typical for amorphous bodies or biological objects. Note that under conditions of TEM experiments (deep vacuum and electron beam impact) these products instantaneously disintegrated, leaving behind a ‘skeleton’ of DND particles—which could be the reason why in earlier studies the *original* fibers were presumed to consist entirely of DND particles. 

Further DWF studies showed that its content changes under different treatments, see Table 7 [85]. The C/N ratio decreases both with longer exposure to air (entries 2 and 3) and with more intense exposure to nitrogen (entries 5 and 6). For more protracted exposures, see Table S1.

This decrease in the C/N ratio in DWFs can have two causes: either the already existing nitrogen impurities from DND are somehow concentrated in DWF, or the fixation of atmospheric nitrogen (i.e., conversion of nutritionally inaccessible N_2_ molecule into ammonia and/or nitrate) occurs. The nitrogen content increases with the duration of exposure DW to air; it also increases when air is replaced by pure N_2_ gas. These facts speak in favor of nitrogen fixation from the air. Finally, a replacement of DND with nitrogen-free synthetic diamond (AS) prepared under static high pressure (see entry 8 in Table 7) also gave rise to the formation of DWFs in aqueous colloids [86]. It has been reported that N_2_ can be photo-catalytically reduced to NH_3_ on nanodiamond under UV irradiation, which causes the release of electrons from a diamond surface into water [87]. With this in view, we also applied UV irradiation, but without significant effect (entries 6 and 7) [86]. 

In nature, the fixation of N_2_ is performed mainly by nitrogenases in bacteria, where N_2_ is activated on the MoFe-protein and reduced to ammonia on the Fe-protein [88]. The conversion of N_2_ to HNO_3_ occurs naturally via a reaction of N_2_ with O_2_ in the atmosphere, facilitated by lightning [89]. The photo-driven disproportionation of N_2_ in water

(5) N_2_ + H_2_O → NH_3_ + HNO_3_

was achieved under ambient conditions using visible-light irradiation and Fe-doped TiO_2_ as the catalyst, where the chemisorbed N_2_ molecules are activated by oxygen vacancies associated with the Fe centers [90].

Alternatively, is a direct reaction between diamond carbon and molecular nitrogen

(6) 2C + N_2_ = C_2_N_2_

thermodynamically possible? If we take the atomization energy of carbon as 716.7 kJ/mol (that of bulk diamond), then Δ*H* = 309 kJ/mol, i.e., the reaction is highly unfavorable under standard conditions and only possible above 1400 °C [91]. However, it would be exothermic for carbon in the form of atoms or C_2_ molecules, with Δ*H* = −1124.5 or −506.5 kJ/mol, respectively. It is well known that the *E_a_* of nanoparticles is substantially lower than that of the corresponding bulk materials and decreases further as the particle size decreases [75,76,77,92], i.e., the share of surface atoms with lower coordination numbers increases. Note that the heat of combustion (from DSC) of DND is elevated by 30% compared to 100 μm grains of HPHT. Then the reaction

(7) C_2_N_2_ + 2H_2_O + ½ O_2_ = NH_4_NO_3_ + 2C

can become exothermic and the formation of ammonium nitrate is thermodynamically justified. Considering Reactions (6) and (7) together, carbon acts as a catalyst for the formation of ammonium nitrate; note that catalytic properties of DND in other reactions were reported earlier [93,94]. The details of our experiments for obtaining NH_4_NO_3_ will be published in [95].

**Figure 7:**
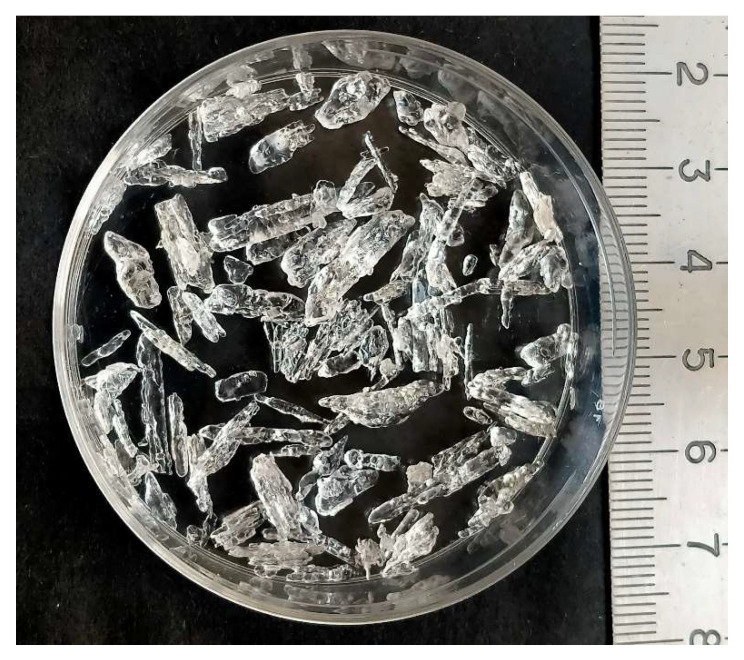
Crystals of ammonium nitrate (phase IV) synthesized by reaction of DW with air.

An amazing feature of this process is the formation of the final product in the form of large crystal grains of NH_4_NO_3_ (see Figure 7) as a result of a very long (1.5 years) contact of DND particles in colloidal solution with air. 

A very small concentration of these particles, which served as centers of crystallization of the formed NH_4_NO_3_, remained in the composition of transparent NH_4_NO_3_ crystals, as shown by chemical analysis (C, 0.22; H, 5.10; N, 33.75; O 60.93%). The slow kinetics of these reactions is explained by the same low concentration of nanodiamond, which is a carrier of fixed nitrogen from air to water. 

It is well known that nitrogen is vital for all living organisms, as an ingredient of proteins and nucleic acids. It is also necessary for production of various industrial chemicals, both large-scale and fine. However, most of the nitrogen on Earth is in the form of chemically inert N_2_ molecules in the atmosphere. The ‘fixation’ of atmospheric nitrogen, which involves breaking up the extremely strong N≡N bond, and its conversion into chemically useable compounds, e.g., ammonia or nitrogen oxides, is thus a major challenge in chemistry. The importance of fixation, which has been carried out by the Haber–Bosch method for more than 100 years, is evident from the huge scale of global ammonia production (170 million tons per year), which accounts for up to 1–2% of the global energy consumption. The high cost, high energy costs and environmental harm (the release of a huge amount of carbon dioxide into the atmosphere) of this method make us look for alternatives, to which up to 30,000 publications and citations for the topic of ammonia synthesis have been devoted annually [96]. 

## 4. Biological Properties of DND Colloids

Bacterial and fungal spores are omnipresent in air and infestation of water exposed to it is only a matter of time; however, we have shown that an admixture of nanodiamond, even in small concentration, greatly facilitates such infestation and subsequent growth in bacterial and fungal colonies. The effects of DND on living cells have been studied extensively, but usually with the focus on biocompatibility, i.e., the absence of cytotoxicity. It has been shown that DND is biocompatible with a broad variety of eukaryotic cells [5,97,98], but depending on its surface composition, DND can also exhibit bactericidal properties [99,100]. The possibility of DND *encouraging* cellular (including bacterial and fungal) growth has not attracted attention it deserves, given the practical applications of DND in medicine (drug delivery, biosensors, etc.).

As mentioned above, in vials of DW exposed to atmospheric air at room temperature, after some periods of time, cloudy and then cotton wool-like fibers (DWF) were observed forming. In identical vials of pure water kept alongside, no fibers were formed; moreover, if transplanted there, the fibers failed to develop and soon disintegrated. The IR spectra of DWFs were very similar to those of typical fungi [101]. Biochemistry tests (aniline blue and lactophenol cotton blue) also indicated biological nature of DWFs [84].

**Figure 8:**
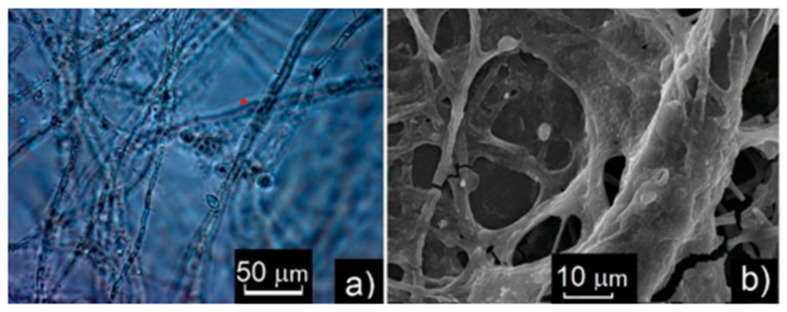
Optical microscopy (**a**) and SEM (**b**) images of NDF, revealing fungal hyphae and spores.

Both SEM and optical microscopy [86] showed DWF to be an entangled fungal mycelium (Figure 8) with fungal spores and separate bacterial cells (bacilli- and cocci-like) entangled in the hyphae, attached to their surfaces or free-floating in DW. The diameters of the mycelial hyphae were characteristic for fungi (1.5 to 18.0 μm, vs. typical spore sizes of 2.5 to 4.5 μm), whereas those of the bacterial cells varied in the usual range (0.2–2.0 μm). The fungi growing in DW formed conidiophores with individual spores. The cocci had the diameters of 0.25 to 0.75 μm, while the bacilli were 1 to 2 μm wide and 2 to 4 μm long. The fungal species identified included *Penicillium cyclopium*, *P. aurantiogriseum*, *P. aurantiogriseum*, *P. chrysogenum*, *Purpureocillium lilacinum*, *Beaveria bassiana*, some species of the genera *Mucor*, *Rhizopus*, *Trichoderma* and *Aspergillus*, as well as some unidentified species. Various bacterial mucous colonies were observed, as well as colonies characteristic of *Bacillus mycoides*, without full identification.

Inoculation with the original DND powder did not produce the growth and formation of colonies of fungi or bacteria after 10 days, even though individual bacteria and fragments of mycelia were present. Inoculation with original DND exposed to air and moisture for a short time, already after 1 week yielded isolated colonies of *Penicillium* bacteria, particularly *P. cyclopium*, clear white mycelium, and *B. mycoides*; on the third week, fungal colonies evolved, distinguished by clear dark-colored mycelium.

Neither fungi nor bacteria were isolated using DND previously sterilized in an autoclave (at 1 atm and 394 K), whether immediately before the tests or after kept hermetically sealed for 1 year. Even after a protracted (1 month) incubation of sterile DND in sterile water, fungi or bacteria did not emerge or grow. If the original DND was placed into sterile distilled water, 1 month later some isolated fungal hyphae and bacterial cells were detected. If a sterile DND powder was incubated in ordinary distilled water or a distilled water previously exposed (for 1 to 3 h) to atmospheric air, then the growth in fungal mycelia and bacterial cells was also observed. Note that blank experiments (pure water samples kept next to DW under the same conditions) never produced DWF, and that mature DWF quickly dies if transplanted from DW to pure water. 

The elemental analysis of the evolving DWF (comprising fungal mycelia with attached bacteria and entrapped DND, which were practically impossible to disentangle) showed a wide variation of their chemical composition (wt%, for the dried material): C, 40 to 74, N, 1.5 to 2.9, H, 1.9 to 3.4, the balance (presumably O), 21.7 to 56.5. The absolute and relative content of carbon and nitrogen in DWF samples generally agrees with that in fungal mycelia and spores, or in bacteria. In micromycetic mycelia the nitrogen content varies from 1.3% to 11% [102]. With the carbon content of 44 to 57%, the C/N ratio in fungi can vary from 4.5:1 to 43:1, the most common ratio being ca. 10. In bacteria the nitrogen content (4–15%) is, on average, higher and the C/N ratio lower (3:1 to 10:1) than in fungi [103].

The evolution of the DWF composition is instructive (see Table S1). The C/N ratio in *growing* fibers slowly decreased from 25 to 11 in the first 10 weeks of incubation (cf. 40 in pure DND), then dropped from 4 to <1 after the 12th week. The latter stage is probably the lysis of micro-organisms, whereupon carbon is released as CO_2_ and nitrogen as ammonia or nitrates. 

The growth in fungi and formation of fibrous and/or fluffy DWF was observed also after bubbling N_2_ gas through DW. Remarkably, N_2_-bubbling for 24 h gave a lower C/N ratio (10.7) than air bubbling under identical conditions (14.5). Mycelial fungi are known which can develop not just under anaerobic, but even under anaerobic *and* oligotrophic conditions [104,105]. However, similar fungal growth in water in the presence of other nanoparticles has not been observed. 

Alongside the fibers, we observed peculiar cylindrical features resembling hollow tubes sealed on both ends, the nature of which was not established. X-ray spectral analyses gave the composition of the tubular features as C 64.7 and O 16.6, cf. C 75.8 and O 4.2 wt% for the surrounding DWFs, or C 86.3 and O 4.7 wt% for initial DND powder particles. No other elements with Z > 5 was detected; although the sum total of elements found was below 100%, this can be largely a spurious effect due to the porosity of the samples and lack of a flat horizontal surface. The highest oxygen content of up to 19.3 wt% was found in one of the ‘tubes’ (15 µm wide and 120 µm long). As soon as the electron beam was directed at such a tube, a red flash (observed by optical microscopy) appeared inside it and quickly propagated along the entire tube, converting it into ash (C 74.3 and O, 1.3 wt%), i.e., combustion in vacuum occurred, which was remarkable. Similar reaction was observed in DND, where the presence of oxygen makes it a micro-explosive. Indeed, compression of DND powder by Bridgman anvils caused a spontaneous detonation when the pressure reached 23–25 kbar, the smoke produced having the characteristic smell of nitric oxides [85]. 

## 5. Conclusions

The experimental study of the physical, chemical and biological behaviors of clear diluted aqueous colloids of DND led to unexpected results which cannot be explained by known theoretical models.

Thus, although the giant DP of confined water on other solids is known, a sharp increase in the DP on heating of DND colloids, and a simultaneous increase in the particle sizes, is completely unexpected. This result rules out the orientation polarization as the cause of giant DP of the solution, since re-orientation of larger DND particles is more difficult. 

The lattice of the DND particles in aqueous solutions, directly observed in a luminescent microscope due to the fluorescence of nanodiamond grains, indicates inter-particle distances in µm range, which are consistent with the distances calculated from the concentrations of DND in DW. The largest particles in these solutions eventually precipitate due to Stokes’ law, that leads to the restructuring of lattices, i.e., to phase transitions in colloids and changes in their electro-physical properties. 

Giant DP of the DND colloids occurs only in polar solvents and persists in aqueous colloids even after dilution by 10^8^–10^10^ times, but is completely absent in non-polar solvents, e.g., CCl_4_.

The increased chemical activity of nanosized diamond converts the reaction of carbon with N_2_ into an exothermic process, allowing the fixation of atmospheric nitrogen, yielding crystalline NH_4_NO_3_ or sustaining bacterial and fungal growth in DND colloids. These results are very important for the ongoing search for new methods of fixation of atmospheric nitrogen under mild conditions, to replace the energy-intensive Haber–Bosch process. The catalytic properties of DND can thus be harnessed to reduce the energy footprint of our civilization while helping to feed the Earth’s growing population. 

**Figure 9:**
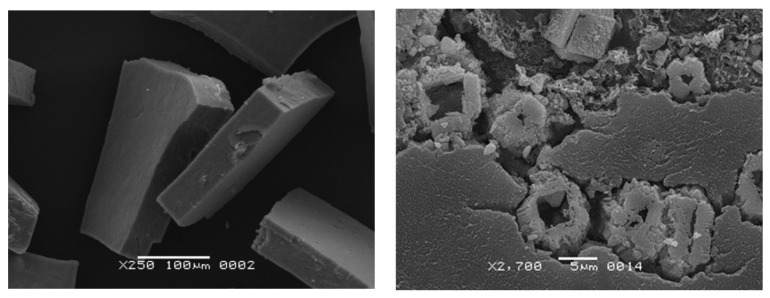
Solid products of evaporation of the DW in sterile conditions.

This issue is also relevant to one of the most fundamental, as well as fascinating, scientific problems: the origin of life on Earth. The importance of water/solid surfaces was first considered by Bernal [106], and this idea later was developed by Szent-Györgyi [107,108] and others [109,110,111,112,113,114] who suggested that life could have started with confined water inducing order to prebiotic molecules on solid surfaces. Indeed, it was suggested recently that impact diamonds from meteorites [115,116], if they became exposed to water, could form the earliest organisms. Since DWF comprises living organisms, the very fact of its formation under oligotrophic conditions is of particular interest in this regard. Obviously, it is impossible to expect the formation of such complex structures as in bacteria and fungi at the first stage of prebiotic synthesis, but the difference between the characteristic shapes of DWF formed under ambient and sterile (Figure 9) conditions, established by us recently, is a good incentive for further research. It is noteworthy that the formation, development and evolution of chemical composition of DWF could be effectively studied only in clear, low-concentration colloidal solutions, although some sporadic observations of them had been reported earlier.

The peculiarities of physico-chemical and biological properties of DND, reviewed above, are principally due to size effect. Evidently, the smaller the nanodiamond particles, the more manifest this effect would be. However, the observed size distributions in both the dry state and in water, as discussed above, show that in as-produced DND, the particles with the size of ≤5 nm are naturally scarce, which significantly reduces its usefulness for research and technological applications. To reduce the particles sizes, or to increase the proportion of de-agglomerated primary particles, three routes have been suggested, viz., (a) milling with balls (beads) of ever-smaller sizes, to maximize the pressure on impact [117]; (b) removing the surface layers of the particles by controlled oxidation [31]; and (c) varying the composition of the explosives and the procedure purification, i.e., of extracting DND from the detonation soot. Given the extreme hardness of diamond, the first approach does not allow to break up the diamond core itself, while contaminating DND with the bead material. The second approach thickens the oxygen-containing coat around the diamond core, which can mask the size effect of nanodiamond and prevent the direct contact of carbon and nitrogen required for nitrogen fixation. Therefore, the work of our team focused on the third approach, which size utilizes the dependence of the particle size on the composition of the explosive [118] and homogeneity of the detonation cloud, i.e., the mixture of the gaseous detonation products [119]. The latter comprises, besides carbon, mainly H_2_O, N_2_, CO, CO_2_, and smaller amounts of H_2_, O_2_, NO, NO_2_, and NH_3_ molecules. Therefore, the probability of just five C_2_ molecules meeting to form a 10-atom diamond cluster is 1/5^9^, i.e., practically nil [120]. On the other hand, when we used benzotrifuroxan (C_6_N_6_O_6_) as the explosive, thus excluding hydrogen (as well as water and ammonia) from the detonation cloud, the size of the DND diamond core increased fivefold [121].

Development of an effective technique for obtaining and preserving de-aggregated primary DND particles of the smallest size would be crucial for the technological prospects of clear DND colloids in physical, chemical and biological applications.

## Figures and Tables

**Table 1:** Radii of particles in DW of different sizes and concentrations.

*D*, μm	*V*_p_, μm^3^	*C*, vol%	*V*_w,_ μm^3^	*R*_w_, μm	*C*, vol%	*V*_w,_ μm^3^	*R*_w_, μm
0.05	5.24 × 10^−4^	0.045	1.164	1.06		0.1164	0.488
0.10	4.19 × 10^−3^		9.311	2.10	0.45	0.9311	0.976
0.20	3.35 × 10^−2^		74.44	4.21		7.444	1.95
0.40	2.68 × 10^−1^		595.5	8.41		59.55	3.90

**Table 2:** Dielectric properties of DND colloids in organic solvents.

Solvent	CCl_4_	PhCl	ClCH_2_CH_2_Cl	Me_2_CO
μ of the solvent, in D	0	1.95	2.27	2.88
ε of the solvent (±1%)	2.24	5.70	10.4	20.7
ε of the colloid (±1%)	2.28	6.9	23	88

**Table 3:** Electro-physical properties of DW vs. DND concentration [27].

DND, wt%	0.000	0.017	0.023	0.034	0.057	0.170
ε, 1 kHz	78.5	4.75 × 10^3^	6.47 × 10^3^	1.40 × 10^4^	4.86 × 10^4^	1.41 × 10^5^
R, kΩ	10^3^	0.139	0.129	0.093	0.060	0.033

**Table 4:** Sizes (nm) of DND agglomerates on dilution.

Dilution Factor	Min. D	Mean D	Max. D
1 ^a^	164.2	223.8	342.0
10	105.7	211.3	396.1
100	105.7	245.1	531.2
1000	32.67	187.0	531.2

^a^ the initial colloid, 0.017 wt% of DND.

**Table 5:** Coordination numbers and atomization energies (kJ/mol) in bulk elemental solids vs. their 5 nm particles.

M	Cu	Au	C	Si	Ge	Mn	Fe	Co	Ni	Pt
*N*_b_	12	12	4	4	4	8	8	12	12	12
*N*_p_	10.52	10.33	3.63	3.44	3.41	6.86	6.96	10.28	10.55	10.40
*E*_b_	337.4	368.2	716.7	450.0	372.0	659.0	415.5	426.7	430.1	565.7
*E*_p_	295.8	317.0	650.4	387.0	317.1	565.1	361.5	365.5	378.1	490.3

**Table 6:** Compositions and properties of dried DWFs.

Samples	Elemental Analysis, %				ρ, g cm^−3^	Refractive Index
	C	N	H	O		
1	40.1	1.5	1.9	56.5	1.88	1.64
2	55.0	2.8	3.2	39.0		
3	67.4	2.9	3.4	26.3	1.92	1.67
4	74.1	2.4	1.8	21.7	2.28	1.78

**Table 7:** Composition (wt%) and C/N weight ratios of the residues from dried DND colloids.

№	DND	Treatment	C	H	N	O	C/N
0	powder		87.1	0.5	2.2	10.2	40.7
1	colloid	Dried at 60 °C, 4 h	86.0	0.3	2.2	11.6	40.0
2	colloid	Air-dried, 1 week	69.6	1.2	4.2	25.0	16.6
3	colloid	Air-dried, 3 weeks	42.6	3.0	3.4	51.1	12.7
4	colloid	Air-bubbled, 24 h	52.1	1.3	3.6	43.0	14.5
5	colloid	N_2_-Bubbled, 24 h	51.2	1.3	4.8	42.7	10.7
6	colloid	N_2_-Bubbled, UV, 11 h	27.2	1.8	1.3	69.6	20.8
7	colloid	N_2_-Bubbled, dark, 11 h	34.4	1.9	2.0	61.7	17.2
8	AS colloid	N_2_-Bubbled, 2 weeks	35.0	1.5	2.4	61.1	14.9

## Supplementary Materials

The following supporting information can be downloaded at: https://www.mdpi.com/article/10.3390/ma16186227/s1, Table S1: composition of dried DWF, depending on their age.
