# Supplementary material for: Transparent Colloids of Detonation Nanodiamond: Physical, Chemical and Biological Properties
Source: Materials (Basel). 2023 Sep 15;16(18):6227. doi: 10.3390/ma16186227 (PMC10532683; doi:10.3390/ma16186227)
Supplement: Supplementary file 1 [file materials-16-06227-s001.zip › materials-2562793-supplementary.pdf]

Supplementary Information for

Transparent Colloids of Detonation Nanodiamond: Physical, Chemical and Biological Properties

Stepan S. Batsanov, *, Sergey M. Gavrilkin, Dmitry A. Dan'kin, Andrei S. Batsanov, Alexander V. Kurakov, Tatiana B. Shatalova and Inna M. Kulikova

Table S1. Composition of dried DWF, depending on their age

Age, weeks	Sample	Elemental analysis, %				C/N ratio
		C	N	H	O	
0[a]	DND	86.5	2.15	0.5	*ca.* 10	40.2
2-3	1	40.1	1.6	1.95	56.35	25.1
	2	67.4	2.9	3.45	26.25	23.2
	3	27.2	1.3	1.8	69.7	20.9
	4	55.0	2.8	3.2	39.0	19.6
4-5	5	34.4	2.0	1.9	61.7	17.2
	6	69.6	4.2	1.2	25.0	16.6
	7	35.0	2.4	1.5	61.1	14.6
	8	52.1	3.6	1.3	43.0	14.5
6-9	9	42.6	3.4	3.0	51.0	12.5
	10	51.2	4.8	1.3	42.7	10.7
10-12	11	10.6	2.5	2.7	84.2	4.24
	12	4.7	2.2	2.1	91.0	2.14
13-16	13	15.7	14.9	3.0	66.4	1.05
	14	2.9	6.85	2.95	87.3	0.42
	15	3.6	13.5	4.3	78.6	0.27
	16	2.65	17.8	5.3	74.25	0.15

[a] Initial DND, dried at 150°C

Atomic composition of 4 samples with the smallest C/N ratio can be rationalized as:

$$C_{1.3}\,N_{1.05}\,H_{2.3}\,O_{4.15} \approx H_2O\cdot 2CO_2\cdot NH_4NO_3$$

$$C_{0.24}\,N_{0.49}\,H_3\,O_{5.45} \approx 4\,H_2O\cdot CO_2\cdot NH_4NO_3\cdot 4O_3$$

$$C_{0.3}\,N_1\,H_{4.3}O_{4.9} \approx 4\,H_2O\cdot CO_2\cdot NH_4NO_3\cdot NO_2$$

$$C_{0.22}\,N_{1.27}\,H_{5.3}\,O_{4.64} \approx 4\,H_2O\cdot C_{1/3}\cdot NH_4NO_3$$
